# 

**Published:** 1878-11

**Authors:** 


					﻿Whole Number,
CCCCII.
November, 1878.
Number 5,
Vol. XXXVII.
THE
CHICAGO
MEDICAL
T ournal and Examiner
EDITORS:
WILLIAM H. BYFORD, A.M., M.D.,
JAS. NEVINS HYDE, A.M., M.D. FERD. C. HOTZ, M.D.
E. FLETCHER INCALS, M.D.
CHICAGO:
PUBLISHED BY THE CHICAGO MEDICAL PRESS ASSOCIATION.
188 Clark Street.
;g"Read the Notice of this Journal among Advertisements.
				

